# Peeping into Human Renal Calcium Oxalate Stone Matrix: Characterization of Novel Proteins Involved in the Intricate Mechanism of Urolithiasis

**DOI:** 10.1371/journal.pone.0069916

**Published:** 2013-07-24

**Authors:** Kanu Priya Aggarwal, Simran Tandon, Pradeep Kumar Naik, Shrawan Kumar Singh, Chanderdeep Tandon

**Affiliations:** 1 Department of Biotechnology and Bioinformatics, Jaypee University of Information Technology, Waknaghat, Solan, Himachal Pradesh, India; 2 Department of Urology, Post Graduate Institute of Medical Education & Research (PGIMER), Chandigarh, India; UAE University, Faculty of Medicine & Health Sciences, United Arab Emirates

## Abstract

**Background:**

The increasing number of patients suffering from urolithiasis represents one of the major challenges which nephrologists face worldwide today. For enhancing therapeutic outcomes of this disease, the pathogenic basis for the formation of renal stones is the need of hour. Proteins are found as major component in human renal stone matrix and are considered to have a potential role in crystal–membrane interaction, crystal growth and stone formation but their role in urolithiasis still remains obscure.

**Methods:**

Proteins were isolated from the matrix of human CaOx containing kidney stones. Proteins having MW>3 kDa were subjected to anion exchange chromatography followed by molecular-sieve chromatography. The effect of these purified proteins was tested against CaOx nucleation and growth and on oxalate injured Madin–Darby Canine Kidney (MDCK) renal epithelial cells for their activity. Proteins were identified by Matrix-assisted laser desorption/ionization-time of flight (MALDI-TOF MS) followed by database search with MASCOT server. *In silico* molecular interaction studies with CaOx crystals were also investigated.

**Results:**

Five proteins were identified from the matrix of calcium oxalate kidney stones by MALDI-TOF MS followed by database search with MASCOT server with the competence to control the stone formation process. Out of which two proteins were promoters, two were inhibitors and one protein had a dual activity of both inhibition and promotion towards CaOx nucleation and growth. Further molecular modelling calculations revealed the mode of interaction of these proteins with CaOx at the molecular level.

**Conclusions:**

We identified and characterized Ethanolamine-phosphate cytidylyltransferase, Ras GTPase-activating-like protein, UDP-glucose:glycoprotein glucosyltransferase 2, RIMS-binding protein 3A, Macrophage-capping protein as novel proteins from the matrix of human calcium oxalate stone which play a critical role in kidney stone formation. Thus, these proteins having potential to modulate calcium oxalate crystallization will throw light on understanding and controlling urolithiasis in humans.

## Introduction

Human renal stones are composed of crystalline and non-crystalline phases; 80% of stones are composed of calcium oxalate (CaOx) and the supporting structure i.e. the organic matrix accounts for 2–5% of the total stone weight [1], [2] and is distributed throughout the architecture of all stones [3]. Proteins constitute a major portion of the matrix and the organic matrix is considered to be important in stone formation and growth [4]. Macromolecules are suggested to direct the course of crystallization by inducing crystal nucleation on the surface and acting as an adhesive or bridge for the binding of crystals together to form large aggregates and in providing a platform for the deposition of more solute, thereby leading to crystal growth [5].

Under physiological conditions urinary supersaturation with CaOx is never high enough to result in homogenous nucleation; a promoter is likely to contribute to the precipitation of this salt [6]. Pure promoters of urolithiasis are rare, but some substances can act as promoters at particular stages of crystal formation and as inhibitors at other stages, e.g. glycosaminoglycans promote crystal nucleation but inhibit crystal aggregation and growth. Tamm-Horsfall glycoprotein (THP), depending on its stage of aggregation, may act as a promoter or an inhibitor of crystal formation [7]. Several proteins have been detected in human stone organic matrix [8], [9] till now but their involvement in stone formation is still not known. Stone research has come a long way to achieve the current ideas regarding stone pathogenesis at molecular levels, but still the mechanism behind stone formation remains obscure. Hyperoxaluria has been stated as the predisposing factor for stone formation [10]. Research has identified that two-third of oxalate accumulates in the cytoplasm of renal cells under pathological conditions, suggesting that the oxalate may play a pivotal role in disturbances at the molecular level [11]. Oxalate-mediated gene expression has also been well documented, and the overexpression of lithogenic proteins [12], crystal-binding molecules such as osteopontin occurs during hyperoxaluric conditions [13], [14]. Proteins that can bind to oxalate would be mediators of such pathologic expression. Hence, identification of such proteins can throw light on stone pathogenesis. Present studies were conducted to isolate proteins from the human renal stone matrix and to assess their influence on different stages of CaOx formation. Herein, we present evidence for the presence of five novel proteins from human kidney stone matrix which play a critical role in influencing stone formation.

## Materials and Methods

### Human Renal Stones Collection

Approval for the present study was obtained from Institutional Ethical Committee of Post Graduate Institute of Medical Education and Research (PGIMER), Chandigarh, India (Dated: 25/11/2011; No: PGI/IEC/2011/560-561). Participants provided their verbal informed consent to participate in this study. A record was made of the patients who gave their consent for use of their surgically removed stones. The ethics committees of Post Graduate Institute of Medical Education and Research, approved this consent procedure. Stones were of non-infectious nature and were collected from those patients who were more than 25 years of age and were suffering from no other abnormality. After Fourier transform infrared spectroscopy (FTIR) analysis, the stones with calcium and oxalate as their major components were selected and used in present study. Stones from each patient was not treated separately and were collected from different patients and were pooled together to conduct this study. Fifty stones with calcium and oxalate, as the major components were used for further studies. Fifty stone samples were randomly pooled into 5 groups, each group containing 10 stone samples.

### Protein Extraction from Human Renal Stones

Proteins were isolated from the matrix of kidney stones containing CaOx as the major constituent using EGTA as a demineralizing agent. Stones were washed in 0.15 M NaCl with gentle stirring for 48 h to remove the adhered blood, tissue etc. They were then dried and pulverized with a mortar and pestle. The powdered stone organic matrix was then extracted by suspending each gram of stone in 10 mL of 0.05 M EGTA, 1 mM PMSF and 1% β-mercaptoethanol. The extraction was carried out for 4 days at 4°C with constant stirring. The suspension was centrifuged for 30 min at 10,000 x g and at 4°C. The supernatant of EGTA extract was filtered through Amicon ultra centrifugal filter device with 3kDa molecular weight cut off at 4°C and concentrated it. Whole EGTA extract and greater than 3 kDa fractions were stored at −20°C for further studies [15].

### Protein Determination

Total protein concentration was determined by Lowry’s method using BSA as a standard [16].

### Assay to Measure Inhibitory Activity of Protein w.r.t CaOx Crystal Nucleation

The method used was similar to that described by Hennequin et al. with some minor modifications [17]. Solutions of calcium chloride (CaCl_2_) and sodium oxalate (Na_2_C_2_O_4_) were prepared at the final concentration of 3 m mol L^−1^ and 0.5 m mol L^−1^, respectively, in a buffer containing Tris 0.05 mol L^−1^ and NaCl 0.15 mol L^−1^ at pH 6.5. Both solutions were filtered through a 0.22 µm filter; 1.5 mL of CaCl_2_ solution was mixed with different concentrations of extracted proteins. Crystallization was started by adding 1.5 mL of Na_2_C_2_O_4_ solution. The final solution was stirred at 37°C repeatedly after an interval of 60 sec for 8 min. The absorbance of the solution was monitored at 620 nm after every 60 sec. The percentage inhibition produced by the protein extract was calculated as [1-(Tsi/Tsc)] X 100, where Tsc was the turbidity slope of the control and Tsi the turbidity slope in the presence of the inhibitor.

### Assay to Measure Activity of Protein w.r.t. CaOx Crystal Growth

Activity against CaOx crystal growth was measured using the seeded, solution-depletion assay [18]. Briefly, 1.5 mg/ml of CaOx crystal (from FTIR identified clinical kidney stones) slurry was added to a solution containing 1 mM CaCl_2_ and 1 mM Na_2_C_2_O_4_. The reaction between CaCl_2_ and Na_2_C_2_O_4_ would lead to deposition of CaOx on the crystal surface leading to the depletion in free oxalate that is detectable by spectrophotometer at λ214 nm. When a protein is added into this solution, the rate of depletion of free oxalate will decrease if the protein inhibits CaOx crystal growth. Rate of reduction of free oxalate was calculated using the baseline value and the value after incubation with or without protein. The relative inhibitory activity was calculated as follows: % Relative inhibitory activity = [(C–S)/C]×100, where C is the rate of reduction of free oxalate without any test protein and S is the rate of reduction of free oxalate with a test protein.

### SDS PAGE

MINI-PROTEAN 3 cell (Bio-Rad Laboratories) was used for SDS-PAGE analysis. Lyophilized samples were reconstituted in reducing sample buffer and analyzed by one-dimensional discontinuous SDS-PAGE using 1 mm thick, 10% separating and 4.4% stacking gels. Protein bands were stained with silver using ProteoSilver™ Plus Silver Stain Kit (PROTSIL2, Sigma-Aldrich Co.). Broad range and low range molecular weight markers (catalog # 161-0317, # 161-0304 Bio-Rad) were used as standards [19].

### NATIVE PAGE

The electrophoresis of the fraction obtained after molecular sieve chromatography was performed maintaining the native configuration of protein biomolecules. The gel for native page was used in the concentration of 10% without using SDS or 2-mercaptoethanol (reducing agent). The molarity of electrophoresis buffer used for native gels was 50 mM Tris-Cl and 284 mM glycine. The gels were over run for 15 min and were stained using silver staining.

### Cell Culture

Madin-Darby Canine kidney (MDCK) cells were obtained from National Centre of Cell Sciences (NCCS, Pune). The cells were maintained as monolayers in Dulbecco’s Modified Eagle’s Medium (DMEM) adjusted to contain 3.7 g/L sodium bicarbonate, 4.5 g/L glucose and 2.0 mM L-glutamine. Media was supplemented with 1% Penicillin (100 units/mL)-Streptomycin (10,000 µg/mL) and 10% fetal bovine serum. Cells were cultured in 25 cm^2^ tissue-culture treated flasks at 37°C and 5% CO_2_ in humidified chambers [20].

### Oxalate-induced Cell Injury

MDCK cells were incubated in DMEM containing 1 mM sodium oxalate in the presence of different concentrations of protein samples for 72 h [21], [22]. Cell injury was assessed by measuring the cell viability by tetrazolium (MTT) colorimetric assay, sulphorhodamine B (SRB) protein stain assay and Acridine Orange/Ethidium Bromide (AO/EB) Staining to Detect Apoptosis.

### Preparation of the Protein Samples

For cell culture studies, the proteins were dialyzed through Millipore Amicon Ultra Centrifugal Filters (3 kDa) and desalted by Bio-Rad ReadyPrep 2-D Cleanup Kit and was reconstituted in distilled water filtered using Millipore Millex GV Filter Unit 0.22 µm. This was treated as a stock solution of the proteins.

### Cell Culture Studies: MTT Assay

The MDCK cells were seeded into two 96-well plates (1×10^4^ cells/well). Following plating and a 24 h recovery for the cells to resume exponential growth at 37°C in a humidified incubator containing 5% CO_2,_ the cells were subjected to various treatments_._ Cells of the control group were cultured in 100 µl of DMEM medium only (without FBS). For oxalate injury, cells were treated with 1 mM oxalate in DMEM. The effect of the proteins in the presence of oxalate injury was assessed by adding purified proteins at various concentrations (1, 2, 4 µg/ml) in the presence of oxalate, to the cells. To verify whether the purified proteins by themselves contributed to cytotoxicity, cells were also incubated with the various concentrations (1, 2, 4 µg/ml) of the proteins in the absence of oxalate. After 72 h of treatment, 30 µl of MTT (final concentration of 0.5 mg/ml) was added to each well. Three hours later, the supernatant was discarded and acid isopropanol was added to dissolve the formazan crystals. Absorbance values were determined at a 570 nm test wavelength and a 630 nm reference wavelength to test the cell viability using a microplate reader (Bio-Rad). [23].

### Cell Culture Studies: SRB Assay

Sulphorhodamine (SRB) assay was performed as described previously in the literature [24]. The seeding and treatment schedules were the same as that for MTT assay. After 72 h of treatment, cells were fixed by means of protein precipitation with 40% ice-cold tricholoroactetic acid (50 µl/well, final concentration 1%) for 1 h at 4°C. After fixation, cells were washed for five times with distilled water and were stained for 30 min. with 0.4% SRB dissolved in 1% acetic acid (100 µl/well) at room temperature. The unbound SRB dye was removed by washing the plates five times with 1% acetic acid. The plates were air dried and the bound protein stain was solubilised with 200 µl/well of 10 mM unbuffered Tris Base (pH 10.5). The optical density was read at 490 nm using a Bio-Rad 3350 micro-plate reader.

### Acridine Orange/Ethidium Bromide Staining To Detect Apoptosis

0.5 ×10^ 5^cells/well of MDCK was seeded in a 24 well plate and was incubated for 24 h in a humidified incubator containing 5% CO_2_. Cells of the blank control group were cultured in DMEM medium (without FBS) only. For oxalate injury cells were treated with 1 mM oxalate in DMEM. Cells were treated with purified proteins at concentration of 4 µg/ml in the presence of oxalate. Also the effect of only the purified proteins was also tested to check for its cytotoxic effects on cells. After 72 h of treatment, the cell suspension from each well along with cells after trypsinization were pooled together into eppendorf vials. The vials were centrifuged at 129 g for 5 min. The pellet obtained was washed with 1X PBS and stained with Acridine Orange/Ethidium Bromide solution of 25 µL PBS and 2 µL EB/AO dye (100 µg/ml). The cells were place on a glass-slide covered with cover-slips and were visualized under the fluorescence microscope (Nikon EclipseTi) at a magnification of 200X. The following parameters were taken into consideration while capturing photographs: Excitation wavelength and Emission wavelength used for acridine orange was 440 nm to 480 nm and 520 nm to 560 nm, while Excitation wavelength and Emission wavelength for ethidium bromide was 445 nm and 605 nm [25].

### Purification and Characterization of Proteins

More than 3 kDa EGTA fraction exhibited significant activity on CaOx crystal nucleation and growth assay system, therefore it was subjected to strong anion exchanger Macro Prep® High 25 Q (Bio-Rad). The column (50X1cm) was previously washed and equilibrated with 20 mM Tris buffer with 0.1 mM NaCl (pH 7.4). Bound proteins were eluted by incorporating a linear concentration gradient of NaCl (0.1–1 M) in the column buffer while keeping the pH constant at a flow rate of 0.5 mL/min. All fractions were monitored for protein content (A280) and simultaneously their conductivity was measured. Fractions coming under the peak were pooled, dialyzed and their bioactivity was studied. All the fractions obtained were concentrated and loaded one by one on a Bio gel® P-100 gel molecular sieve column (50×1 cm) equilibrated and eluted with the 20 m mol L^−1^ Tris buffer (pH7.4) at a flow rate of 0.1 ml/min [26]. The fractions which eluted out based on their molecular weights were pooled to study their activity w.r.t. CaOx crystal nucleation and growth as well as on oxalate induced injury on MDCK cells.

### RP-HPLC for Homogeneity

Waters Spherisorb® C18 (5 *μ*, 4.6 X 250 mm) column with solvent A (0.1% TFA in water) and solvent B (100% acetonitrile containing 0.1% TFA) was used for determining the homogeneity of purified protein. Flow rate was maintained at 1 mL/min at the time of protein injection. The column was washed with solvent A and brought to 20% acetonitrile in 5 min. The bound protein was eluted with a linear gradient of acetonitrile (20–70%) over a period of 50 min. The detection was monitored at 280 nm using Waters 2996 photodiode array detector [27].

### Tryptic In-gel Digestion of Purified Protein

Single band detected after molecular-sieve chromatography was excised from the gel and was destained with destainer provided in the ProteoSilver™ Plus Silver Stain Kit (PROTSIL2, Sigma-Aldrich Co.). Trypsin profile IGD kit (PPO100, Sigma-Aldrich Co.) was used for in-gel digestion of purified protein. Destained gel piece was dried for approximately 15 to 30 min. Trypsin solubilised in 1 m mol L^−1^ HCl and mixed with 40 m mol L^−1^ ammonium bicarbonate and 9% acetonitrile was added to the destained gel piece. Gel piece was fully covered by the addition of 40 m mol L^−1^ ammonium bicarbonate and 9% acetonitrile (pH 8.2) solution and was incubated for 5 h at 37°C. After the incubation, liquid was removed from the gel pieces and transferred to fresh Eppendorf tubes and was preserved for mass spectroscopic analysis.

### Peptide Mass Fingerprinting by MALDI-TOF-MS and Sequence Analysis

Each proteolytic sample was premixed 1∶2 with the matrix solution (α-Cyano-4-hydroxycinnamic acid) and spotted on the sample stage. It was dried at room temperature followed by washing with 0.1% TFA and was analyzed by Ultraflex TOF/TOF mass spectrometer (Bruker Daltonics, Remen, Germany). Mass spectrometer was calibrated by peptide calibration standard II (Bruker). Acquired mass spectra had resolution of ∼6000 (FWHM), which was sufficient to identify the digested peptide. The mass/charge spectra obtained were searched in MASCOT search engine (http://www.matrixscience.com) using all the 3 databases (MSDB, SwissProt, NCBInr). For search, peptides were assumed monoisotopic, oxidized at methionine residues and carbamidomethylated at cysteine residues. A Homo sapiens taxonomy restriction was used, only one missed cleavage was allowed, and peptide mass tolerance of 1.2 kDa was used for peptide mass fingerprinting.

### Homology Modeling and Structure Validation

Homology model building of the identified proteins: ethanolamine-phosphate cytidylyltransferase and macrophage-capping protein was done using molecular operating environment (MOE) [28], [29]. The template used for model building of ethanolamine-phosphate cytidylyltransferase was the crystal structure of human CTP: Phosphoethanolamine Cytidylyltransferase in complex with CMP [PDB ID: 3ELB_A] with a query coverage of 85% and sequence identity of 98%. Similarly the crystal structure of mutant macrophage capping protein (Cap G) with actin-severing activity in the Ca^2+^ free form [PDB ID: 1J72_A] was used as a template for model building of macrophage-capping protein with a query coverage of 100% and sequence identity of 95%. The structure obtained were energy minimized using OPLS 2005 force field with Polak-Ribiere Conjugate Gradient (PRCG) algorithm. The minimization was stopped either after 5,000 steps or after the energy gradient converged below 0.001 kcal/mol. All atom molecular dynamics (MD) simulation of protein structures in explicit water was carried out using the GROMACS 4.5.4 software and the GROMOS96 force field for a time scale of 10 ns [30]. Three-dimensional periodic boundary conditions were imposed, enclosing the molecule in a dodecahedron solvated with the SPC216 water model provided in the GROMACS package and energy minimized using 1000 steps of steepest descent. The LINCS [31] algorithm was used to constrain all bond lengths and cutoff distances for the calculation of the coulombic and Van der Waals interactions at 1.0 nm. The system was equilibrated by 100 ps of MD runs with position restraints on the protein to allow the relaxation of the solvent molecules at 300 K and normal pressure. The overall quality of the model, stereochemical values and non-bonded interactions were tested using PROCHECK [32], ERRAT [33] and VERIFY3D [34].

However, no suitable templates were found for the proteins: Ras GTPase-activating-like protein, UDP-glucose:glycoprotein glucosyltransferase 2 and RIMS-binding protein 3A to build their structures.

### Docking Molecular of Calcium Oxalate

Calcium oxalate structure was built and geometrically optimized with the help of molecular builder of Molecular Operating Environment (MOE) package developed by the Chemical Computing Group Inc. Montreal, Canada. Binding sites of proteins were predicted by using active site finder tool of MOE software. Molecular docking of calcium oxalate was done using MOE–Dock. MOE-dock utilizes a Monte Carlo Simulated Annealing (SA) method in docking calculations to search for favorable binding configurations of a small, flexible ligand and a rigid macromolecule in a pre-set box. The docking energy calculation was carried out within a user-specified three-dimensional docking box (3D docking box) using the simulated annealing method and OPLS-AA force field. The energy grids for docking were generated as grid-based potential fields by the MOE-Dock program. Docking energy was calculated as the sum of the electrostatic, Van der Waals, and flexibility energies. The Van der Waals parameters were taken from the force field. The electrostatic field was calculated based on force field in the Coulombic manner using the constant dielectric of 1.0 for solvation. MOE-Dock performed 25 independent docking runs and the lowest docking energy conformation for each binding site was chosen for LIGPLOT analysis [35].

### Molecular Interaction of Calcium Oxalate and Purified Proteins

To investigate the precise interaction between calcium oxalate and proteins Ethanolamine-phosphate cytidylyltransferase and Macrophage-capping protein identified in this study we have mutated the amino acids in the binding site. Precisely all the acidic and basic amino acids were mutated to alanine and the amino acids such as Tyrosine, Threonine and Serine in the active site were phosphorylated. After incorporating these mutations, calcium oxalate was docked into the binding site using MOE-Dock with similar parameter as was used for wild type.

### Statistical Analysis

The experiments were performed in triplicate. The data are presented as means ± SD. Statistical evaluation was conducted by one-way ANOVA using Tukey's multiple comparison. A minimum P value <0.05 was considered to be the minimum level of statistical significance. The software used for analysis was GraphPad InStat. Where ‘*’represents P values versus untreated control and ‘#’represents P values versus oxalate control. Where **** P<0.0001, *** P<0.001, ** P<0.01, * P<0.1; and #### P<0.0001, ### P<0.001, ## P<0.01, # P<0.1.

## Results

### Bioactivity-guided Purification of Proteins with Potential to Influence CaOx Crystal Nucleation and Growth

**Figure 1:**
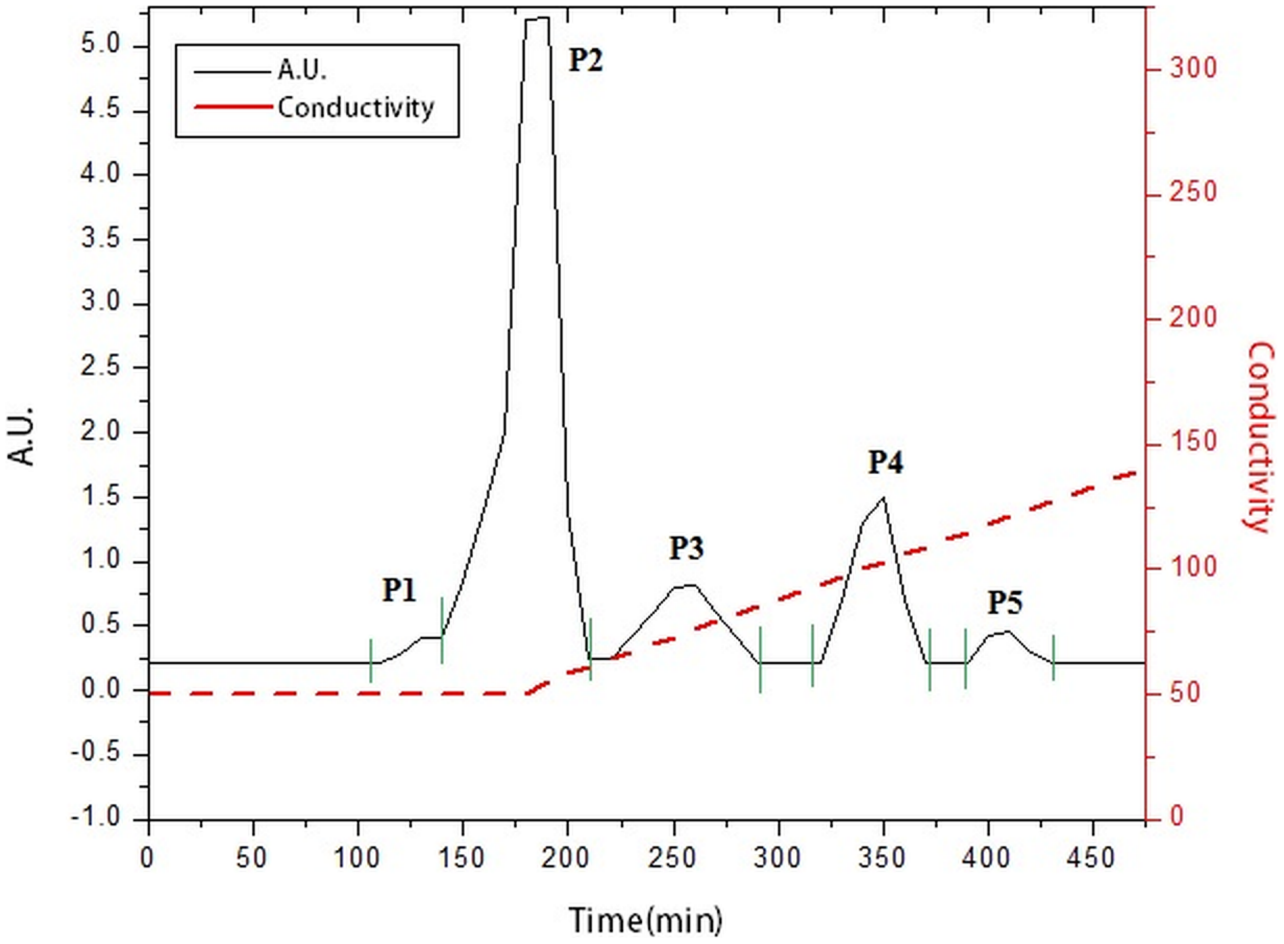
Elution profile of anion exchange chromatography. Elution profile of >3kDa protein sample loaded on anion exchanger. P3, P4 and P5 fractions were collected with a linear gradient of NaCl.

**Figure 2:**
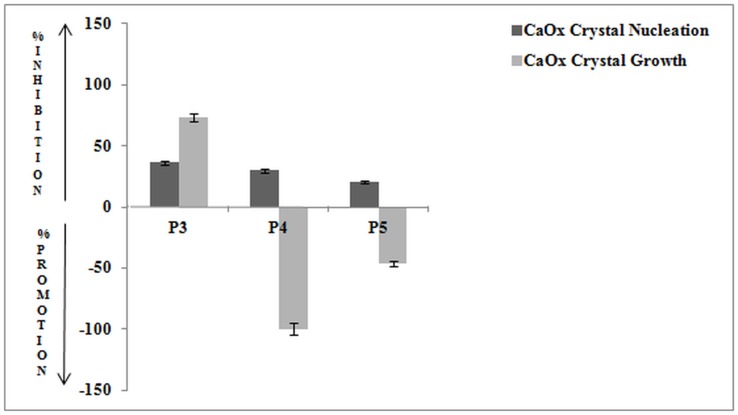
Bioactivity of eluted fractions. Percentage activity of pooled fractions of anion exchange chromatography against CaOx crystal nucleation and growth assay.

**Figure 3:**
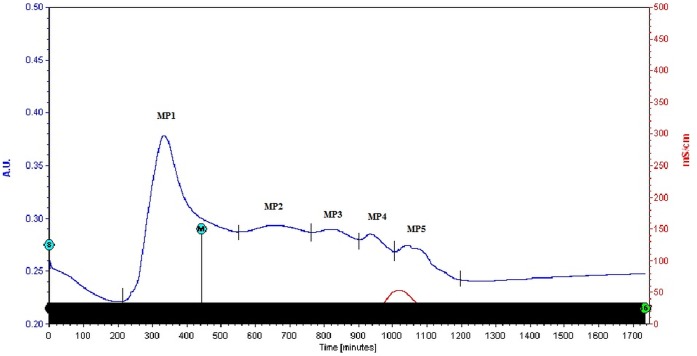
Purification by molecular sieve chromatography. Elution profile of fraction P3 loaded on molecular-sieve chromatography column after anion exchange chromatography.

**Figure 4:**
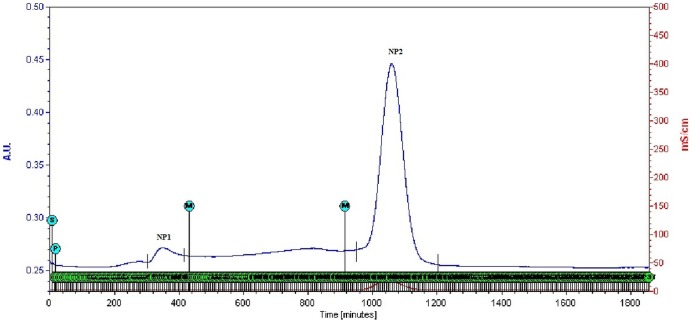
Purification by molecular sieve chromatography. Elution profile of fraction P4 loaded on molecular-sieve chromatography column after anion exchange chromatography.

**Figure 5:**
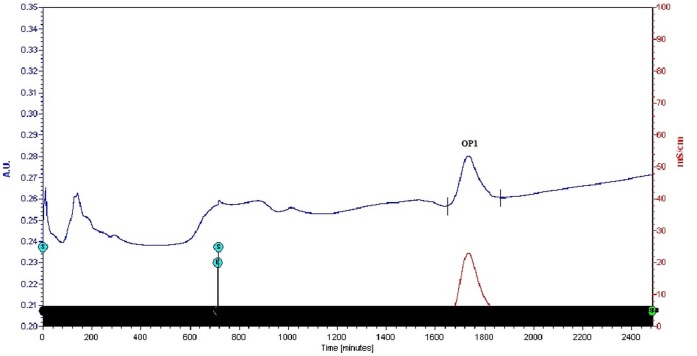
Purification by molecular sieve chromatography. Elution profile of fraction P5 loaded on molecular-sieve chromatography column after anion exchange chromatography.

**Figure 6:**
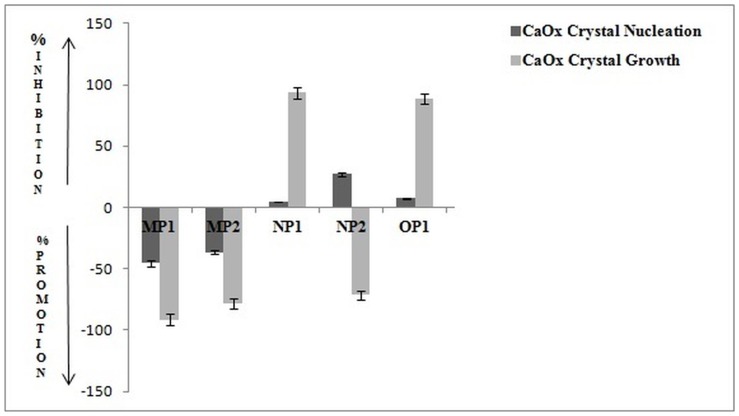
Bioactivity of purified fractions. Percentage activity of most potent fractions of molecular-sieve chromatography against CaOx crystal nucleation and growth assay.

Whole EGTA extract, greater than and less than 3kda fractions were assayed to measure activity against CaOx crystal nucleation and growth. Whole EGTA extract as well as greater than 3kDa fraction exhibited significant inhibitory activity as compared to the less than 3kDa fraction. Greater than 3kDa fraction exhibited significant activity towards calcium oxalate monohydrate nucleation and growth assay system and hence was selected for purification purpose. More than 3kDa fraction was loaded on a strong anion exchanger Macro Prep® 25 Q column. Consecutive fractions were collected with increasing gradient, pooled and were named P3 to P5 (Figure 1). It was found that the three fractions P3 to P5 exhibited inhibitory activity against CaOx crystal nucleation, whereas P3 exhibited inhibitory activity and P4, P5 exhibited promoter activity towards CaOx crystal growth (Figure 2). Further SDS-PAGE of P3, P4, P5 analysis showed presence of few bands. Fractions P3, P4, P5 were further purified individually by molecular sieve chromatography (Figure 3, 4, 5) on a Bio gel® P-100 gel molecular sieve column (50 X 1cm). Proteins were eluted at pH 7.4 with 20 mM Tris buffer. Fractions collected after purification of the fraction P3 by molecular sieve chromatography were pooled as MP1 to MP5. Fractions collected after purification of P4 by molecular sieve chromatography and were pooled as NP1 & NP2 and purification of P5 by molecular sieve chromatography and were pooled as OP1. The fractions exhibiting the highest activity against CaOx crystal nucleation and growth are represented (Figure 6). These Fractions were further analyzed by 10% SDS-PAGE.

**Figure 7:**
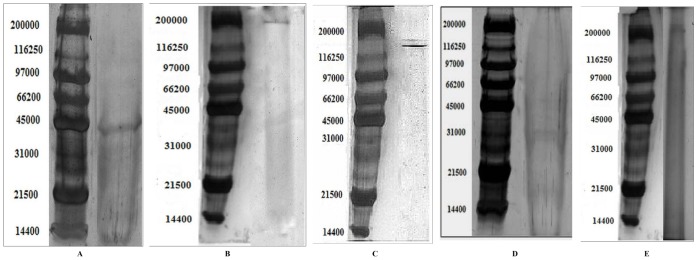
SDS PAGE. SDS PAGE of pooled fractions after molecular sieve chromatography.

SDS –PAGE of MP1 showed the presence of single band of molecular weight ∼44 kDa (Figure 7A). SDS-PAGE of MP2 showed the presence of 5 bands of molecular weight in the range of 30 kDa to 45 kDa, whereas Native PAGE of MP2 revealed the presence of a single band of molecular weight of ∼181 kDa (Figure 7B). SDS-PAGE of NP1 showed the presence of 5 bands of molecular weight in the range of 21 kDa to 45 kDa, whereas Native PAGE of NP1 revealed the presence of a single band of molecular weight of ∼175 kDa (Figure 7C). SDS –PAGE of NP2 showed the presence of single band of molecular weight of ∼38 kDa (Figure 7D). SDS-PAGE of OP1 showed the presence of 6 bands of molecular weight in the range of 21 kDa to 45 kDa. Native PAGE of OP1 revealed the presence of a single band of molecular weight of ∼183 kDa (Figure 7E).

### Mass spectrometric Identification of Novel Proteins

**Figure 8:**
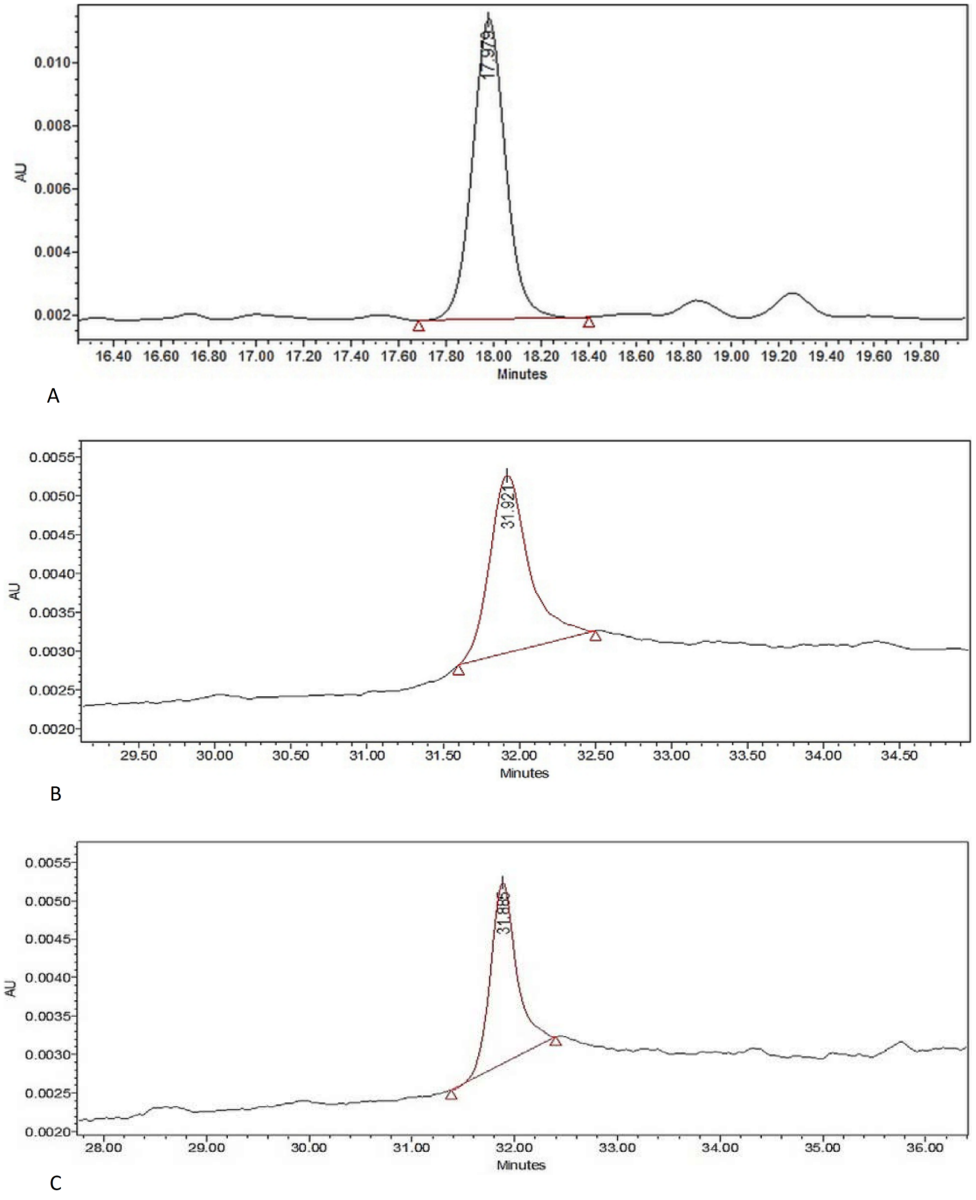
HPLC analysis. HPLC analysis of (A) MP1; (B) MP2; (C) NP1.

**Figure 9:**
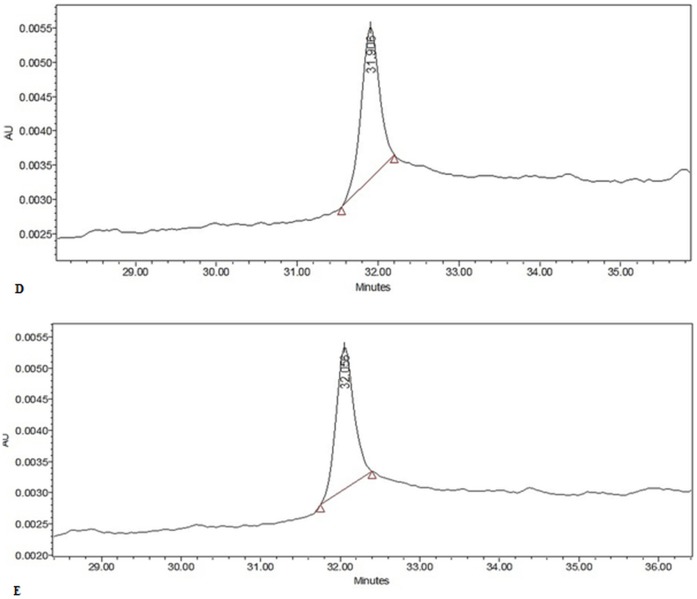
HPLC analysis. HPLC analysis of (D) NP2; (E) OP1.

The protein bands detected in fractions MP1, MP2, NP1, NP2 and OP1 were excised, in-gel trypsin digested and identified by matrix assisted laser desorption/ionization–time of flight (MALDI-TOF) MS. Purity of the samples was tested using RP-HPLC (Figure 8, 9). Using the Mascot search engine (http://www.matrixscience.com), the MALDI-TOF data obtained from fractions MP1, MP2, NP1, NP2, OP1 matched significantly with Ethanolamine-phosphate cytidylyltransferase, Ras GTPase-activating-like protein, UDP-glucose:glycoprotein glucosyltransferase 2, Macrophage-capping protein and RIMS-binding protein 3A respectively (Table 1). MASCOT search engine revealed that these proteins had MOWSE score of 26, 31, 23, 26 and 23 respectively and with good sequence coverage.

**Table 1:** Identified proteins using the Mascot search engine (http://www.matrixscience.com), from the data obtained from MALDI-TOF of the fractions MP1, MP2, NP1, NP2, OP1.

	Matching Score	Sequence Coverage	Molecular weight (Da)	pI
Ethanolamine-phosphate cytidylyltransferase	26	26%	44272	6.44
Ras GTPase-activating-like protein	31	12%	181046	5.47
UDP-glucose:glycoprotein glucosyltransferase 2	23	8%	175262	6.43
RIMS-binding protein 3A	23	11%	183041	6.46
Macrophage-capping protein	26	11%	38765	5.82

### Reduction of Oxalate-induced Renal Tubular Epithelial Cell Injury by Purified Protein (MTT ASSAY)

**Figure 10:**
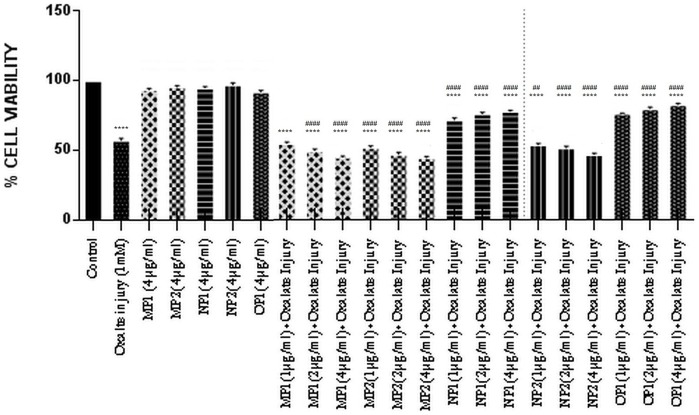
Bioactivity of purified proteins on MDCK cells (MTT assay). Effect of purified proteins from human renal stone martix on MDCK viability analysed by MTT assay. Data are mean ± SD of three independent observations. Where ‘*’ represents P values versus untreated control and ‘#’ represents P values versus oxalate control. Values are the mean and SD of each group (n = 3). All treatments groups were simultaneously compared via one-way ANOVA using Tukey multiple comparison test. Where **** P<0.0001, *** P<0.001, ** P<0.01, * P<0.1; and #### P<0.0001, ### P<0.001, ## P<0.01, # P<0.1.

The effect of the purified proteins was assessed towards the oxalate injured renal tubular epithelial cells, MDCK (Figure 10). Cells were exposed to varying concentrations of purified proteins in the presence of oxalate for 72 h. Cells treated with culture medium only served as untreated control group. The cell viability was expressed as a percentage relative to the untreated control cells. In the cells injured on exposure to oxalate, the cell viability was significantly decreased from 100% in the untreated cells (control) to 55.95%. Treatment of cells with protein alone did not lead to any alteration in cell viability w.r.t. control. Exposure to various concentrations of the purified proteins OP1 and NP1, in the presence of oxalate, resulted in a significant increase in cellular viability in a dose dependent manner. Whereas the purified proteins MP1, MP2, NP2, in a dose dependent manner led to a decrease in cell viability, thereby revealing that these proteins enhance the injury caused by oxalate to the cells.

### Reduction of Oxalate-induced Renal Tubular Epithelial Cell Injury by Purified Protein (SRB ASSAY)

**Figure 11:**
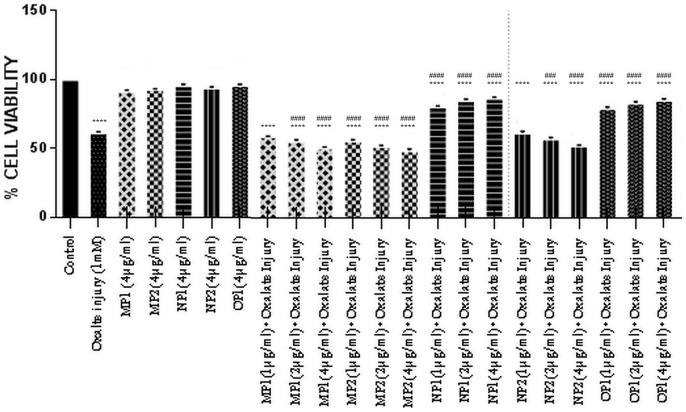
Bioactivity of purified proteins on MDCK cells (SRB assay). Effect of purified proteins from human renal stone martix on MDCK viability analysed by SRB assay. Data are mean ± SD of three independent observations. Where ‘*’ represents P values versus untreated control and ‘#’ represents P values versus oxalate control. Values are the mean and SD of each group (n = 3). All treatments groups were simultaneously compared via one-way ANOVA using Tukey multiple comparison test. Where **** P<0.0001, *** P<0.001, ** P<0.01, * P<0.1; and #### P<0.0001, ### P<0.001, ## P<0.01, # P<0.1.

A similar pattern of cell viability was observed with SRB assay (Figure 11), which confirms the activity of the different purified proteins towards the oxalate injured renal cells. In SRB assay the cell viability was expressed as a percentage relative to the untreated control cells. In the cells injured with exposure to oxalate, the percentage cell viability was significantly reduced from 100% in the untreated cells (control) to 61.23% on exposure to oxalate. Treatment of cells with protein alone did not lead to any alteration in cell viability w.r.t. control. The cell viability was significantly increased in the cells which had been exposed to different concentrations of the purified proteins (OP1 & NP1) along with oxalate in a concentration dependent manner, revealing the protective nature of the proteins towards the injured oxalate cells. The results for MP1, MP2, NP2 reiterated the findings seen in the previous assay for viability, that various proteins behave differentially in the presence of injury towards the renal cells.

### Acridine Orange/Ethidium Bromide Staining To Detect Apoptosis

**Figure 12:**
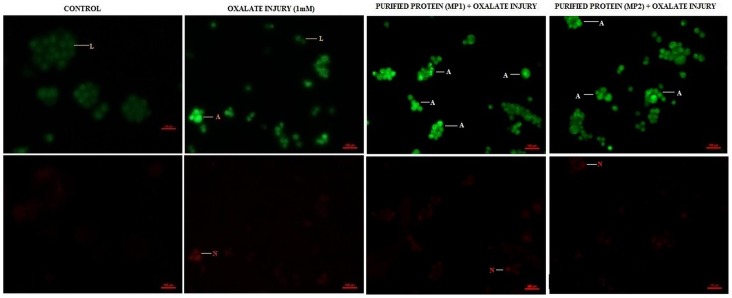
Bioactivity of purified proteins on MDCK cells (AO/EtBr). Effect of purified proteins MP1 and MP2 from human renal stone matrix on MDCK cells by AO/EtBr staining analysis of the induction of apoptosis in MDCK, visualized under fluorescence microscopy at magnification 200X. Viable cells are shown in green, apoptotic cells in fluorescent green and necrotic cells in orange. Cells were harvested at the indicated times and then stained with acridine orange (AO)/ethidium bromide (EtBr). Symbols and labels are used in the same way as in the figure 12.

**Figure 13:**
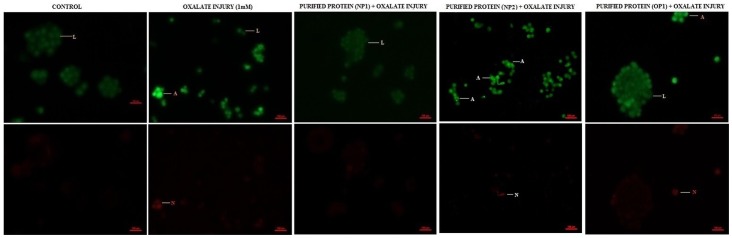
Bioactivity of purified proteins on MDCK cells (AO/EtBr). Effect of purified proteins NP1, NP2 and OP1 from human renal stone matrix on MDCK cells by AO/EtBr staining analysis of the induction of apoptosis in MDCK, visualized under fluorescence microscopy at magnification 200X. Viable cells are shown in green, apoptotic cells in fluorescent green and necrotic cells in orange. Cells were harvested at the indicated times and then stained with acridine orange (AO)/ethidium bromide (EtBr). Symbols and labels are used in the same way as in the figure 13.

Differential staining patterns using a combination of two fluorescent dyes, acridine orange and ethidium bromide, was used to categorise the manner in which cell cytotoxicity was taking place in the cells (Figure 12, 13). Acridine Orange (AO) enters all cells and results in a green appearance of the nuclei. When the cytoplasmic integrity is compromised, ethidium bromide (EB) permeates the cells and stains the nucleus red. This results in an easily distinguishable pattern, wherein live cells have a normal green nucleus; apoptotic cells range from having a bright green to orange nucleus with a condensed and fragmented chromatin; necrotic cells display a structurally normal orange nucleus.

We observed that there was no apoptosis and necrosis in the control i.e. all cells were live. Treatment of cells with protein alone did not lead to any alteration in cell viability w.r.t. control. When cells were injured with oxalate, a substantial level of cell death was observed, i.e. both apoptotic and necrotic. However, cells treated with purified proteins (OP1 & NP1), in the presence of oxalate, showed a reduced level of apoptosis and necrosis w.r.t. the oxalate injured cells. Whereas when the cells were treated with MP1, MP2, NP2 the degree of apoptosis and necrosis was increased w.r.t. the oxalate injured cells. The purified proteins alone, did not result in cell death.

### Homology Modeling of Ethanolamine-phosphate cytidylyltransferase and Macrophage-Capping Protein and Molecular Docking with Calcium Oxalate

**Figure 14:**
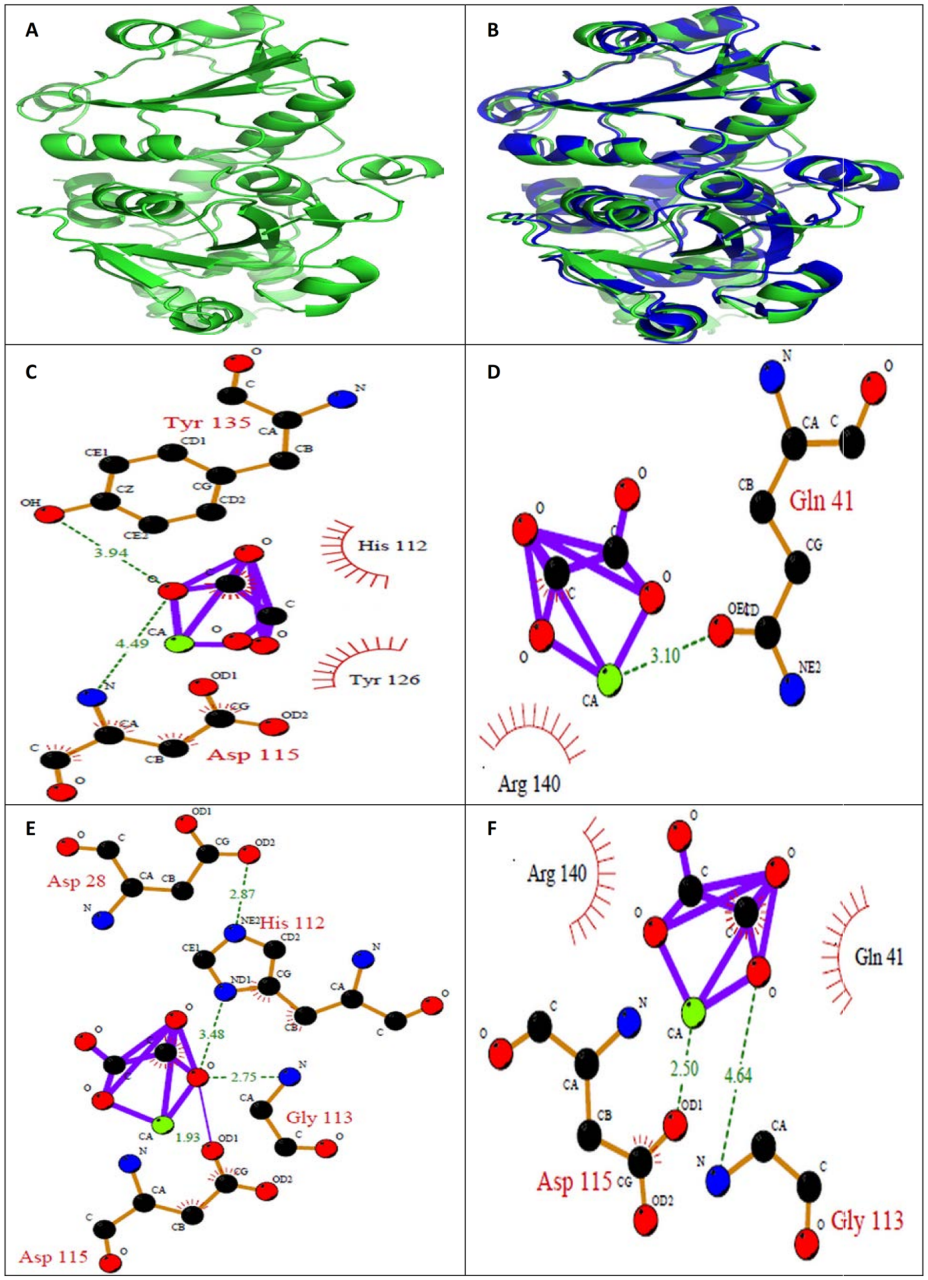
*In silico* analysis of Ethanolamine-phosphate cytidylyltransferase. (A) Homology modeled structure of Ethanolamine-phosphate cytidylyltransferase (B) Superposition of modeled structure and the template of Ethanolamine-phosphate cytidylyltransferase (RMSD value: 0.912); Ligplot of Ethanolamine-phosphate cytidylyltransferase (C) Wild type; (D) M1; (E) M2; (F) M3; where M1: Mutation of acidic amino acid with alanine; M2: Mutation of basic amino acid with alanine; M3: Phosphorylation of tyrosine and threonine. The hydrogen bonds involved in interaction between CaOx and the protein are represented in dotted line.

**Figure 15:**
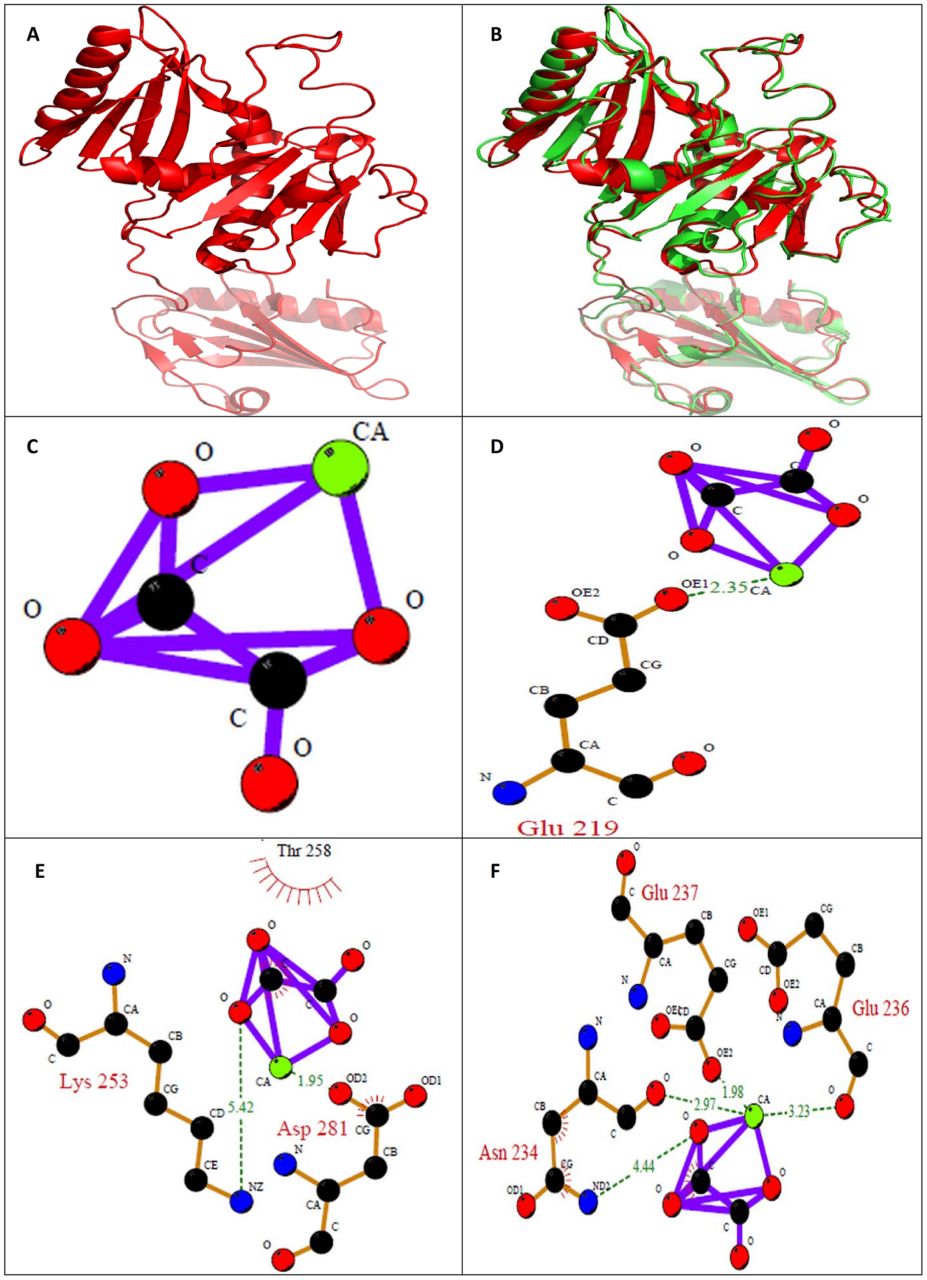
*In silico* analysis of Macrophage-capping protein. (A) Homology modeled structure of Macrophage-capping protein (B) Superposition of modeled structure and the template of Macrophage-capping protein (RMSD value: 1.023); Ligplot of Macrophage-capping protein (C) Wild type; (D) M1; (E) M2; (F) M3; where M1: Mutation of acidic amino acid with alanine; M2: Mutation of basic amino acid with alanine; M3: Phosphorylation of tyrosine and threonine. The hydrogen bonds involved in interaction between CaOx and the protein are represented in dotted line.

The crystal structure of identified proteins: ethanolamine-phosphate cytidylyltransferase (Figure 14) and macrophage-capping protein (Figure 15) was not available in Protein Data Bank which necessitated for developing a protein model. The RMSD between the model structure of ethanolamine-phosphate cytidylyltransferase and the template structure was found to be 0.912 Å. The overall stereochemical quality of the model was assessed by PROCHECK. The Ramachandran plot showed 80.8% residues in most allowed region and 16.8% in additional allowed region. There were no residues in disallowed region and 2.4% in generously allowed region. These results revealed that the majority of the amino acids are in a phi-psi distribution that is consistent with a right-handed α-helix, and the model is reliable and of good quality. The overall main-chain and side-chain parameters, as evaluated by ProCheck, are all very favorable. The Ramachandran plot characteristic confirms the quality of the predicted model. The assessment with VERIFY3D, which derives a “3D–1D” profile based on the local environment of each residue, described by the statistical preferences for the area of the residue that is buried, the fraction of side-chain area that is covered by polar atoms (oxygen and nitrogen) and the local secondary structure, also substantiated the reliability of the three dimensional structure 92.51% of the residues had an average 3D–1D score >0.2.

The crystal structure of identified Macrophage-capping protein was modeled based on the Crystal Structure of Mutant Macrophage Capping Protein (Cap G) with Actin-Severing Activity in the Ca^2+^ -Free form (PDB ID: 1J72_A) of Homo sapiens was used as a template (Figure 15.A). The RMSD between the model structure of Macrophage-capping protein and the template structure was found to be 1.023 Å. The overall stereochemical quality of the model was assessed by PROCHECK. The Ramachandran plot showed 82.5%, residues in most allowed region and 15.8%, in additional allowed region. There were only 0.3% residues in disallowed region and 1.4%, in generously allowed region. These results revealed that the majority of the amino acids are in a phi-psi distribution that is consistent with a right-handed α-helix, and the model is reliable and of good quality. The overall main-chain and side-chain parameters, as evaluated by ProCheck, are all very favorable. The Ramachandran plot characteristic confirms the quality of the predicted model. The assessment with VERIFY3D, which derives a “3D–1D” profile based on the local environment of each residue, described by the statistical preferences for the area of the residue that is buried, the fraction of side-chain area that is covered by polar atoms (oxygen and nitrogen) and the local secondary structure, also substantiated the reliability of the three dimensional structure 77.29% of the residues had an averaged 3D–1D score >0.2.

Different binding sites of the proteins were predicted by MOE Site finder and used for molecular docking. CaOx was docked onto the binding sites using the Monte Carlo docking procedure of MOE. The best-docked poses of the ligand were identified and energy minimized, while allowing full side chain flexibility. Docking of calcium oxalate with the binding site of wild type Ethanolamine-phosphate cytidylyltransferase and Macrophage-capping protein gave best docking score of −8.9374 and −8.8703 kcal/mol respectively (Table 2). In Ethanolamine-phosphate cytidylyltransferase both acidic and basic amino acids seems to be involved in binding with CaOx. This was revealed from the site directed mutation of both acidic and basic amino acids in the binding site. Mutation of acidic amino acids to alanine resulted in decrease of docking score from −8.937kcal/mol to −7.437kcal/mol. Similarly mutating basic amino acids to alanine resulted in decrease in docking score from −8.9374 to −8.3003 kcal/mol. Upon phosphorylation of amino acids the docking score decreased to a value of −8.5647 kcal/mol from −8.9374 kcal/mol indicating involvement of these amino acids. Ligplots were generated to show the interaction between identified proteins and CaOx (ligand). LIGPLOT of ethanolamine-phosphate cytidylyltransferase wild type showed involvement of aspartate (Asp) at position 115 and tyrosine (tyr 135) with calcium oxalate (Figure 14C). Whereas ligplot of ethanolamine-phosphate cytidylyltransferase, when acidic amino acids were mutated showed the involvement Glutamine (Gln 41) with calcium of CaOx (Figure 14D). Amino acids Aspartate (Asp) 115, Glutamine (Gln) 113, Histidine (His) 112, Aspartate (Asp) 28 were found to interact with oxalate of calcium oxalate when basic amino acids were mutated with alanine (Figure 14E). Amino acids Aspartate (Asp) 115 was found to interact with calcium and Glycine (Gly) 113 with oxalate of calcium oxalate upon phosphorylation (Figure 14F).

**Table 2:** Docking scores of Ethanolamine-phosphate cytidylyltransferase and Macrophage-capping protein.

Ethanolamine-phosphate cytidylyltransferase	Docking Score (kcal/mol)	Macrophage-capping protein	Docking Score (kcal/mol)
Wild Type	−8.937 kcal/mol	Wild Type	−8.870 kcal/mol
M1	−7.437 kcal/mol	M1	−6.846 kcal/mol
M2	−8.300 kcal/mol	M2	−7.147 kcal/mol
M3	−8.564 kcal/mol	M3	−8.975 kcal/mol

M1: Mutation of acidic amino acid with alanine; M2: Mutation of basic amino acid with alanine; M3: Phosphorylation of tyrosine and threonine.

In Macrophage-capping protein again both acidic and basic amino acids seem to be involved in binding with CaOx. This was revealed from the site directed mutation of both acidic and basic amino acids in the binding site. Mutation of acidic amino acids to alanine resulted in decrease of docking score from −8.8703 kcal/mol to −6.8467 kcal/mol. Similarly mutating basic amino acids to alanine resulted in decrease in docking score from −8.8703 kcal/mol to −7.1479 kcal/mol. Upon phosphorylation of amino acids the docking score increased to a value of −8.9757 kcal/mol from −8.8703 kcal/mol indicating involvement of these amino acids. LIGPLOT of Macrophage-capping protein wild type did not show involvement of any amino acid with calcium oxalate (Figure 15C). Whereas ligplot of Macrophage-capping protein with mutated acidic amino acids showed the involvement Glutamine (Glu) 219 with calcium of calcium oxalate (Figure 15D). Amino acid Aspartate (Asp) 218 was found to interact with calcium and Lysine (Lys) 253 with oxalate of calcium oxalate when basic amino acids were mutated with alanine (Figure 15E). Amino acids Asparagine (Asn) 234, Glutamine (Glu) 236, Glutamine (Glu) 237 with calcium of calcium oxalate upon phosphorylation (Figure 15F).

The interaction between calcium and acidic amino acids is certainly plausible, but it is equally conceivable that basic residues that are normally protonated at urinary pH and positively charged might experience an attraction toward negatively charged oxalate groups. This is supported by the presence of basic amino acids in the inhibitory proteins. In either case steric constraints from 3D conformation of the molecule might limit the number of these simple interactions. However, no suitable templates were found for the proteins: Ras GTPase-activating-like protein, UDP-glucose:glycoprotein glucosyltransferase 2 and RIMS-binding protein 3A to build their structures.

## Discussion

Proteins present in the organic matrix of renal stones have been proposed to play an important role in kidney stone disease for several decades [36], [37]. Identification of proteins was hampered in the past by limitations in protein identification methods thereby making the identification of novel proteins quite difficult without prior knowledge of their involvement in this process. Recent advances in the technologies are therefore mandatory to study the role played by proteins for better understanding of the pathophysiology and pathogenesis of kidney stone disease. Many proteins present in the organic matrix play an important role in kidney stone formation, but only few of them are identified and fully characterized [27]. The purpose of the present study was to use bioactivity guided protein purification methods and recent advances in mass spectrometric protein identification to characterize and identify novel proteins from the organic matrix of human renal stones having the ability to control the mechanism of stone formation. Stones contain small amounts of embedded proteins which are thought to play an adhesive role in the stone aggregates, and they often are found attached to the tip of renal papilla, presumably through adhesive contacts. [38].

Anionic molecules such as phospholipids, which are embedded in epithelial cell membranes, also are thought to promote the attachment of COM to renal tubules [39]. However, certain urinary molecules are thought by others to suppress crystal aggregation and cell attachment, presumably because of adsorption on COM crystal faces [40]–[45]. The interaction between crystals and renal tubular cells has been proposed to be a crucial event that elicits subsequent cellular responses, leading to kidney stone formation. Cellular responses examined in MDCK cells, during COD crystal adhesion reveal several proteins like metabolic enzymes, cellular structural protein, calcium-binding protein, adhesion molecule, protein involved in RNA metabolism, and chaperone. [46].

A number of crystal binding molecules on surfaces of renal tubular epithelial cells that can promote crystal adhesion have been identified, including CD44 [47], hyaluronan, [47], [48] osteopontin (OPN), [47] annexin II (AnxII), [49] nucleolin-related protein (NRP), [50] and sialic acid-containing glycoprotein. [40], [45]. Sites that bind COM crystals on tubular cells may be only minimally exposed under normal circumstances, but could increase in number following cellular injury, as has been demonstrated for denuded rat bladder urothelium [51], [52]. Certain proteins could mediate adhesion of COM crystals to cells, by altered exposure of crystal binding molecule on the surface of renal tubular cells could promote crystal retention and possibly kidney stone formation [53], [54]. Pre-coating COM crystals with polyanions and pre-coating cells with polycations each inhibited crystal attachment to cultured renal epithelial cells (55), implying that anionic cell surface molecules play a prominent role in attachment of crystals to the cells. Apoptosis of renal tubular cells in response to oxalate and CaOx crystals may play a significant role in CaOx urolithiasis. Khan et al, have suggested that the negatively charged headgroups of phosphatidylserine, which get exposed during apoptosis and membranous cellular degradation products seen during necrosis, attract calcium and can act as sites for attachment of calcific crystals to cell surfaces and thereby can promote crystal nucleation [56], [57].

In the present study, we identified and characterized five novel proteins: Ethanolamine-phosphate cytidylyltransferase, Ras GTPase-activating-like protein, UDP-glucose:glycoprotein glucosyltransferase 2, RIMS-binding protein and Macrophage-capping protein 3A which play a significant role in stone formation. Out of five proteins, two proteins, Ethanolamine-phosphate cytidylyltransferase and Ras GTPase-activating-like protein enhanced the process of stone formation by promoting CaOx crystal nucleation and growth, while two proteins UDP-glucose:glycoprotein glucosyltransferase 2 and RIMS-binding protein 3A inhibited the stone formation process by inhibiting CaOx crystal nucleation and growth while one protein named Macrophage-capping protein exhibited a dual pattern; i.e. inhibition towards CaOx Crystal nucleation and promotion towards CaOx crystal growth.

The interaction between crystals and renal tubular cells has been proposed to be a crucial event that elicits subsequent cellular responses leading to kidney stone formation. Two populations of polyanions, one anchored to the apical plasma membrane and the other free in tubular fluid, can be viewed as competitors for the crystal surface. Alterations in the quality or quantity of either population of anions could alter this competitive balance and thereby determine the ultimate fate of crystals that nucleate from tubular fluid. Crystal adhesion to the tubular cell surface would be favored by an increase in the number of cell surface anionic receptor molecules, a decrease in the quantity of specific anions in tubular fluid, or both.

We identified Ethanolamine-phosphate cytidylyltransferase as a promoter protein which promotes calcium oxalate crystal nucleation and growth. Also, it was observed that this protein enhances the injury caused by oxalate to renal epithilum cells (MDCK) thereby leading to cell death. Ethanolamine-phosphate cytidylyltransferase (EC 2.7.7.14) catalyzes the rate-controlling step in a major pathway for the synthesis of phosphatidylethanolamine (PtdEtn) [58], [59]. S R Khan et al. have reported that calcium oxalate stones contain cardiolipids, sphingomyelin, phosphatidylcholine, phosphatidylionositol, phosphatidylethanolamine, phosphatidylserine, and phosphatidylglycerol in a lipid extract [60], [61]. Endogenous oxalate synthesis makes an important contribution to the amount of oxalate excreted in urine and hence the development of calcium oxalate kidney stones. Lange et al have proposed that glyoxal metabolism may be an important contributor to urinary oxalate excretion. In addition, it has been suggested both glyoxal production and oxalate synthesis can be associated with oxidative stress. The glyoxal can be generated endogenously by various sources including, the catabolism of carbohydrates, proteins, fats and metabolism of glycine, ethanolamine, glycolaldehyde and ascorbic acid. The ethanolamine in turn can be either derived from diet or by the hydrolysis of PE. Therefore, taking cue from the results of our cell viability studies, we thus suggest that this protein; Ethanolamine-phosphate cytidylyltransferase probably plays a crucial role in promoting stone formation, which is also reflected by the presence of PE in the stone lipid content.

The second protein which is Ras GTPase-activating-like protein IQGAP2 is a protein that in humans is encoded by the IQGAP2 gene [62], [63]. The protein contains three IQ domains, one calponin homology domain, one Ras-GAP domain and one WW domain. It interacts with components of the cytoskeleton, with cell adhesion molecules and with several signaling molecules to regulate cell morphology and motility [64]. This protein also associates with calmodulin, a calcium-binding messenger protein which is expressed in all eukaryotic cells. It contains four EF-hand motifs, each of which binds a Ca^2+^ ion [65], [66].Also Ras GTPase-activating-like protein IQGAP2 could serve as an assembly scaffold for the organization of a multimolecular complex that would interface incoming signals to the reorganization of the actin cytoskeleton at the plasma membrane [67]. This protein is expressed in the placenta, lung, and kidney. In our study, we found that Ras GTPase-activating-like protein IQGAP2 promotes promotes calcium oxalate crystal nucleation and growth. Also, it was observed that this protein enhances the injury caused by oxalate to renal epithilum cells (MDCK) thereby leading to cell death. This is in accordance with the fact that this protein contains an IQ domain which interacts with calmodulin which is known to bind with Ca^2+^ ions thereby increasing the probability of stone formation. In addition, by down regulating the ras pathway this protein would lead to decreased cell survival and enhanced apoptosis, which has also been demonstrated in our study.

The third protein was identified as UDP-glucose:glycoprotein glucosyltransferase 2 (UGGT) is an enzyme that resides in the endoplasmic reticulum (ER). UGGT recognizes incompletely folded glycoproteins with N-linked Man9- GlcNAc2 glycans, and transfers a glucose residue from UDP– glucose to produce the Glc1-Man9-GlcNAc2 glycan (68). This monoglucosylated glycan is recognized specifically by the lectin-like chaperones calnexin and calreticulin, which retain incompletely folded glycoproteins in the ER and recruit ERp57 to accelerate disulphide bond interchange for further folding [69], [70], [71], [72]. Higher levels of this enzyme have been found in kidney, pancreas, heart, and skeletal muscle.

It has been observed that urolithiasis can also be a result of misfolding of proteins like,THP [73]. UDP-glucose:glycoprotein glucosyltransferase 2,adds glucose residues to these misfolded proteins allowing calnexin and calreticulin to bind these proteins thereby, preventing their transport from ER to golgi and ultimately sends these proteins for degradation [74], [75]. Therefore, it is probable that this enzyme present in the renal stone matrix could be an adaptive response and thereby prevent further injury. Indeed, we have seen that cells treated with this protein were rescued from oxalate induced injury.

The fourth protein was identified as RIMS-binding protein 3A which belongs to the RIMBP family. RIM-binding proteins (RIM-BPs) were identified as binding partners of the presynaptic active zone proteins RIMs as well as for voltage-gated Ca^2+^ -channels. They were suggested to form a functional link between the synaptic-vesicle fusion apparatus and Ca^2+^-channels. RIM-BP gene family diversified in different stages during evolution, but retained their unique domain structure. RIM-BP3 is exclusively expressed in mammals. All RIM-BP genes encode proteins with three SH3-domains and two to three fibronectin type-III domains. The Fibronectin type III domain is an evolutionary conserved protein domain that is widely found in animal proteins [76]. Fibronectin type III domain has been found to be involved in regulation of wound healing in tumor fibrosis which is considered as a dysregulated wound healing response. Fibronection which has been earlier reported as an inhibitor of stone formation in urolithiasis contains 16 copies of this domain [76]. Thus, we can relate the inhibitory activity of RIMS-binding protein 3A with fibronectin by virtue of having the same domain i.e. Fibronectin type III domain.

The fifth protein viz. Macrophage-capping protein in humans is encoded by the *CAPG* gene found in macrophages and macrophage-like cells. This protein is a member of the gelsolin/villin family of actin-regulatory proteins [77]. It reversibly blocks the barbed ends of F-actin filaments [78] in a Ca^2+^ and phosphoinositide-regulated manner, thus contributes to the control of actin-based motility in non-muscle cells [79], [80]. Macrophages play a role in phagocytosis, or engulf and then digest, cellular debris and pathogens. These cells play a central role in protecting the host and also contribute to the pathogenesis of inflammatory and degenerative diseases. It has been reported that crystals of calcium oxalate monohydrate (COM) that adhere to the cell surface can be internalized by endocytosis [81], [82]. Macrophages and other inflammatory cells can take care of these crystals and destroy them [83]. Large crystal masses with or without insufficient protection by macromolecules will adhere to the tubular cell. The crystals might alter the plasma membrane so that endocytosis occurs; whereas crystals of reasonable size can be taken care of and destroyed by the cell, larger crystal agglomerates might cause cell death [84]. When the crystals that are bound to the basolateral membrane or deposited interstitially are too large, the capacity of macrophages and inflammatory cells will probably be insufficient to cope with the crystals. This protein Macrophage-capping protein was found to have a dual activity wherein on one hand this protein inhibits CaOx nucleation, on the other hand this protein promotes CaOx growth. Such proteins which can play different roles at different stages of stone formation like THP are already reported [7], [85], [86]. In our study, we identified one such protein with dual activity which was found to be present in macrophages; which play a vital role in stone formation.

We focused our attention on the interaction of the purified proteins with calcium oxalate using *in silico* molecular docking studies. *In silico* results revealed that both the acidic and basic amino acids in the binding site are essential for molecular interactions of CaOx with the purified proteins: Ethanolamine-phosphate cytidylyltransferase and Macrophage-capping protein. Whether a protein or another macromolecule acts as an inhibitor of growth and aggregation or a promoter of nucleation and aggregation implies that there must be some mechanism to explain the interaction with the mineral oxalate surfaces. Determining the molecular mechanisms by which urinary constituents modulate calcium oxalate crystallization is crucial for understanding and controlling urolithiassis in humans. Although a few initial molecular-scale investigations of the controlling mechanisms of kidney stone formation by these inhibitory molecules have been performed, the majority of previous studies have been concerned with the overall kinetics of crystallization, rather than molecular mechanisms which remain poorly defined. The interaction between calcium and acidic amino acids is certainly plausible, but it is equally conceivable that basic residues that are normally protonated at urinary pH and positively charged might experience an attraction toward negatively charged oxalate groups. This is supported by the presence of basic amino acids too in the proteins. In either case, steric constraints from 3D conformation of the molecule might limit the number of these simple interactions.

### Conclusions

Nephrolithiasis remains a public health problem around the world, affecting 1–20% of the adult population. Of all types of renal stones, calcium oxalate (CaOx) is the most common composition found by chemical analysis and till date the pathogenic mechanisms of stone formation remain unclear. Proteins are found as major components in human renal stone matrix and are considered to have a potential role in crystal–membrane interaction, crystal growth and stone formation. Recent advances in the technologies are therefore necessary to study kidney stone formation inhibitors for better understanding of the pathophysiology and pathogenesis of kidney stone disease. Few well-characterized inhibitors/promoters of kidney stone formation have been identified earlier.

We identified five proteins namely Ethanolamine-phosphate cytidylyltransferase, Ras GTPase-activating-like protein, UDP-glucose:glycoprotein glucosyltransferase 2, RIMS-binding protein 3A and Macrophage-capping protein as new components of stones from organic matrix of CaOx stones by bioactivity guided-protein purification and MALDI-TOF-MS. The presence of these proteins in the matrix of CaOx stones suggests their putative role in urolithiasis and their relation to the process of stone formation. The crystallization assays used in this study provide sufficient evidence to assert that proteins isolated from renal stone with MW>3 kDa have a tendency to influence crystallization and aggregation of calcium oxalate. Hence, identification of these proteins not only throws light on the intricate mechanism of kidney stone formation but also provides potential drug targets against urolithiasis.
